# Effect of Airborne Particle Abrasion and Nitric–Hydrofluoric Acid Etching on the Mechanical and Structural Properties of 3Y‐ and 5Y‐TZP Zirconia

**DOI:** 10.1155/ijod/3811328

**Published:** 2026-10-13

**Authors:** Yasser M. Aly, Magued H. Fahmy, Ghada Ayash, Ola Taha

**Affiliations:** ^1^ Conservative Dentistry Department Faculty of Dentistry Alexandria University Alexandria Egypt alexu.edu.eg; ^2^ Oral Rehabilitation Sciences Faculty of Dentistry Beirut Arab University Beirut Lebanon bau.edu.lb; ^3^ Faculty of Dentistry Beirut Arab University Beirut Lebanon bau.edu.lb

**Keywords:** 3Y-TZP, 5Y-TZP, acid etching, airborne-particle abrasion, biaxial flexural strength, Zircos E

## Abstract

**Introduction:**

Surface treatment is essential for optimizing zirconia bonding, but conventional airborne‐particle abrasion (APA) may compromise its mechanical properties, particularly in highly translucent materials. Nitric–hydrofluoric acid (HF) etching has recently emerged as a potential alternative, yet its influence on the surface characteristics and mechanical performance of different zirconia generations remains inadequately understood. Therefore, this study evaluated the effects of APA and nitric–HF etching on the surface roughness, crystalline structure, and biaxial flexural strength (BFS) of 3 mol% yttria‐stabilized tetragonal zirconia polycrystal (3Y‐TZP) and 5 mol% Y‐TZP (5Y‐TZP) zirconia.

**Methods:**

Ninety‐six disc‐shaped specimens (10 mm diameter × 1.5 mm thickness) were milled from 3Y‐TZP (ZI) (*n* = 48) and 5Y‐TZP (FX) (*n* = 48). Each zirconia type was randomly allocated into four treatment subgroups (*n* = 12 per subgroup; 8 subgroups in total): (1) untreated control, (2) APA‐50, (3) APA‐120, and (4) Zircos E etching. Surface roughness (Ra) was measured using a Marsurf Ps10 surface roughness analyzer, while the crystalline structure was assessed by X‐ray diffraction (XRD) diffraction and BFS was evaluated using a universal testing machine according to ISO 6872. Data were statistically analyzed using ANOVA (*α* = 0.05), followed by posthoc tests.

**Results:**

APA‐120 showed the highest Ra values (ZI = 1.09 ± 0.29 µm; FX = 0.69 ± 0.12 µm), significantly higher than those of all other treatments (*p*  < 0.001). Acid etching produced Ra values comparable to the controls (ZI = 0.38 ± 0.06 µm; FX = 0.47 ± 0.03 µm; *p*  > 0.05). for XRD, 5Y zirconia remained stable across all treatments, while 3Y zirconia showed slight peak intensity changes and minor tetragonal‐to‐monoclinic transformation after APA, with no change after acid etching. BFS was consistently higher in ZI than in FX (*p*  < 0.001). The highest strength was recorded for untreated ZI (1059 ± 103 MPa), whereas the lowest was observed for FX after APA‐120 (420 ± 80 MPa). Zircos‐E preserved 84.7% of the control strength in ZI (897 ± 153 MPa) and 96% in FX (479 ± 97 MPa), whereas APA‐120 reduced strength by ~20% in 3Y‐TZP and 16% in 5Y‐TZP.

**Conclusions:**

APA with 120 µm Al_2_O_3_ maximizes microroughness but compromises the flexural strength. Zircos E etching produced limited surface roughness changes while preserving crystalline stability and flexural strength in both zirconia generations.

## 1. Introduction

After the amplified usage of CAD/CAM technologies in the dental field, high‐oxide‐content ceramics have been widely used and recommended owing to their esthetic results. The advancements in dental adhesion have also increased the recommendation of bonded restorations [1].

Yttrium‐stabilized tetragonal zirconia polycrystals (Y‐TZPs) can be utilized in a large range of clinical procedures, including fixed partial dentures (FPDs), posts, and cores, or even as implant bodies and abutments for implants. Zirconia is known for its high mechanical properties in comparison to different types of ceramics: 1200 MPa flexural strength, greater than 2000 N of fracture resistance, and finally 9–10 MPa of fracture toughness [2].

Achieving reliable and durable bonding between zirconia and resin cements remains a challenge due to their chemical inertness and lack of a glassy phase. The high particle density and low silica content in zirconia prevent conventional acid etching, limiting effective bonding methods [3].

Airborne‐particle abrasion (APA) is commonly used to improve zirconia bonding by increasing surface roughness; however, it may also induce phase transformation and reduce mechanical strength, especially in highly translucent 5 mol% Y‐TZP (5Y‐TZP) zirconia. Recently, zirconia‐specific acid‐etching systems such as Zircos E have been proposed as conservative alternatives that may enhance surface characteristics while preserving crystalline stability and mechanical integrity. Recent systematic reviews have highlighted the promising potential of chemical etching protocols for zirconia surface treatment, although evidence regarding their effects on different zirconia generations remains limited [4]. It is also essential to evaluate the material’s biaxial flexural strength (BFS) as high surface roughness from certain bonding–enhancing treatments may weaken zirconia’s resistance to fracture under occlusal forces [5, 6].

Zirconia is inherently more opaque than enamel and dentin. To improve its translucency, the yttria content was increased to 5 mol%, producing 5Y‐TZP. Although this modification enhances translucency, it reduces the flexural strength and fracture toughness compared with 3 mol% Y‐TZP (3Y‐TZP) because of the lower proportion of the tetragonal phase [7].

Multiple adhesion protocols have been proposed for zirconia restorations, particularly when mechanical retention is limited [1]. For silica‐based ceramics, conventional hydrofluoric acid (HF) etching, followed by silanization, is the established protocol for achieving durable resin bonding [8]. However, this approach is not directly applicable to zirconia because its polycrystalline, silica‐free structure is resistant to conventional HF etching, limiting its effectiveness for surface conditioning [9]. Although modified acid‐etching protocols have been investigated, their clinical effectiveness remains inconclusive.

Consequently, surface treatment of zirconia restorations is essential to obtain durable adhesion with resin cements. Commonly used approaches include APA with alumina particles to increase surface roughness and silica coating followed by silanization to provide both micromechanical interlocking and chemical coupling [10]. In addition, functional phosphate monomers, particularly 10‐methacryloyloxydecyl dihydrogen phosphate (MDP), have been shown to significantly enhance zirconia bonding when incorporated into ceramic primers or resin cements [11, 12]. MDP promotes chemical adhesion by forming stable phosphate–zirconium bonds with the zirconium oxide layer on the zirconia surface, resulting in improved bond durability and resistance to hydrolytic degradation [13, 14]. A major limitation of air abrasion is the potential reduction in the flexural strength of zirconia due to the formation of surface defects. These defects may propagate over time as a result of low‐temperature degradation (LTD), adversely affecting the material’s mechanical properties. Furthermore, sandblasting with 50‐µm AL_2_O_3_ has been reported to reduce the fracture strength and Weibull modulus of zirconia [5].

To address the difficulty of bonding to zirconia, several studies have investigated HF etching heated to 100°C as a potential surface treatment [15]. However, other studies reported that HF etching may reduce the bond strength between resin cements and zirconia due to the high particle content of this ceramic [16].

Combined nitric–HF etching has also been proposed for zirconia surface treatment. Heated etching with HF for 60 min produced greater surface roughness (103.2 nm) than shorter etching times [17]. However, APA with 50‐µm Al_2_O_3_ achieved higher shear bond strength (8.4 ± 2.7 MPa) than HF etching (3.3 ± 0.6 MPa) [15], and several studies have reported that acid‐etching alone has little or no significant effect on zirconia bonding [18].

Despite extensive research on zirconia surface treatments, evidence regarding the effects of APA and acid etching on the zirconia substrate remains limited and inconsistent. Most studies have focused on resin bond strength rather than changes in the surface characteristics, crystalline structure, and mechanical properties. In addition, comparative data on the response of different zirconia generations, particularly 3Y‐TZP and 5Y‐TZP, to recently introduced nitric–HF etching systems are scarce. Therefore, further investigation is needed to evaluate their effects on both surface modification and the mechanical integrity of zirconia.

The aim of this study was to evaluate the effects of three surface treatment protocols, APA with 50‐µm AL_2_O_3_, APA with 120‐µm Al_2_O_3_, and nitric–HF etching (Zircos E PRO4 Dental) on the surface roughness (Ra), crystalline structure, and BFS of translucent 3Y‐TZP (ZI) and ultratranslucent 5Y‐TZP (FX) zirconia. The null hypothesis was that neither APA nor nitric–HF etching would produce significant differences in the surface roughness, crystalline structure, or BFS of either monolithic zirconia material.

## 2. Materials and Methods

In this in vitro study, Sample size was calculated using G^∗^Power software Version 3.1.9.7, assuming 5% alpha error, 80% study power, and 95% confidence interval [19]. The calculation was based on flexural strength data reported by Elraggal et al. [20], who evaluated the effect of different surface treatments on zirconia. In their study, the mean initial flexural strength ranged from 1236.7 ± 33.3 MPa in the control group to 293.3 ± 38.2 MPa in the FGC + Laser group, while residual flexural strength after fatigue ranged from 950.0 ± 82.7 MPa to 279.8 ± 54.4 MPa. The highest reported standard deviation was 144.0 MPa. Based on these estimates and using a one‐way ANOVA model, a conservative large effect size (*f* = 0.45) was assumed, yielding a minimum required sample size of 10 specimens per subgroup. The calculated sample size was applied to each experimental subgroup in the factorial design, providing sufficient observations for comparisons across both zirconia translucency and surface treatment groups. To compensate for potential specimen loss during processing and testing, the sample size was increased to 12 specimens per subgroup.

### 2.1. Specimen Grouping

A total of 96 specimens were prepared. Group I (ZI) consisted of 48 specimens of 3Y‐TZP zirconia (AmannGirrbach Ceramill ZI), while Group II (FX) consisted of 48 specimens of 5Y‐TZP zirconia (Ceramill Zolid FX, Amann Girrbach AG, Koblach, Austria). Each main group was further subdivided into four subgroups (*n* = 12) according to the surface treatment applied. The control subgroup received no surface treatment. Subgroup APA‐50 was treated with airborne‐particle abrasion using 50 µm aluminum oxide particles. Subgroup APA‐120 was treated with 120 µm aluminum oxide particles using APA. Subgroup Etching was treated using the Zircos E etching system (PRO4 Dental).

### 2.2. Specimen Fabrication

Specimens were first digitally designed with the desired dimensions using computer‐aided design software (AutoCAD; Autodesk Inc.) 10 mm ± 0.2 diameter and 1.5 mm ± 0.2 thickness. Ninety‐six specimens of pri‐sintered yttria‐stabilized zirconia 3Y‐TZP, 3 mol% (AmannGirrbach Ceramill ZI) (*n* = 48) and yttria‐stabilized zirconia 5Y‐TZP, 5 mol% Zolid FX White zirconia (AmannGirrbach AG, Koblach, Austria) (*n* = 48). The standard tessellation language (STL) files were digitally arranged through a nesting protocol and fabricated using a five‐axis dental milling system (Amann Girrbach AG, Motion 2). Zirconia discs (Ceramill ZI and Zolid FX) were individually secured within the milling chamber and processed under dry‐milling conditions. Upon completion of the milling procedure and prior to sintering, each specimen underwent thorough cleaning with pressurized air to remove any remaining debris or milling residues. Afterward, sintering was carried out in a Ceramill Therm high‐temperature furnace (Amann Girrbach AG, Koblach, Austria) according to the manufacturer’s guidelines. For Ceramill ZI, the sintering process involved reaching a temperature of 1550°C with an increasing rate of 100°C/min, maintaining this temperature for 2 h, and then decreasing the temperature at a rate of 100°C/min, as for Zolid FX, the sintering process involved reaching a temperature of 1450°C with 8°C/min, maintaining this temperature for 2 h and then decreasing the temperature at a rate of 8°C/min. The surfaces of the discs were then polished using diamond and silicon carbide abrasive papers ranging from 600 to 1200 µm. Subsequently, the discs were cleaned in distilled water using an ultrasonic machine for 20 s, removed, and left to air dry.

The sintered discs were visually inspected using a stereomicroscope (OLYMPUS 2Z61, Tokyo, Japan) to detect any cracks or defects and were stored in a dry environment until the surface treatments were performed. The dimensions of the specimens were verified after sintering using a digital caliper. The final thickness was confirmed to comply with the target dimensions, and specimens with dimensional deviations beyond the predetermined tolerance (±0.2 mm) were excluded and replaced to ensure thickness uniformity for testing.

### 2.3. Randomization

After specimen fabrication, the zirconia discs were assigned identification numbers and randomly allocated to the four surface treatment groups using a computer‐generated random sequence [21].

### 2.4. Surface Treatments

The sintered specimens were divided into four subgroups according to the surface treatment applied: Control Subgroup: No surface treatment was applied. Subgroup APA 50 µm: Specimens were subjected to APA using 50 µm Al_2_O_3_ particles with a specialized sandblasting unit. The nozzle was positioned perpendicular to the disc surface at a distance of 10 mm for 10 s at 2 bar pressure. To standardize the distance, each disc was placed in a custom‐made cylindrical acrylic mold, ensuring that the nozzle tip was flush with the cylinder opening [22]. Subgroup APA 120 µm: Specimens were treated with 120 µm Al_2_O_3_ particles following the same APA protocol used for Subgroup C [23, 24]. For both APA‐treated groups, the discs were rinsed with distilled water, air‐dried, and ultrasonically cleaned for 3 min to remove any residual particles. Subgroup etching: Specimens were treated with Zircos‐E etchant (PRO4 Dental), a nitric–HF‐based solution. The discs were placed in an incubator at 40°C to maintain the etchant at the specified temperature by the manufacturer for a 30‐m duration. After etching, the specimens were rinsed under cold running water for 2 min to remove the residual acid. All procedures were performed using standard safety precautions, including gloves, masks, and protective eyewear. The etched specimens were air‐dried and stored in a dry environment until testing [23].

### 2.5. Surface Characterization, Crystalline Structure, and BFS Testing

Surface roughness (Ra) was measured at the center of each treated zirconia disc using a Mahr MarSurf PS10 surface roughness analyzer (Mahr Metrology GmbH, Germany), which determines the arithmetic mean surface roughness (Ra) [20]. Prior to specimen testing, the instrument was calibrated using a certified roughness reference standard with a known Ra value in accordance with the manufacturer’s recommendations. An operator blinded to the experimental group allocation performed all measurements. For each specimen, three consecutive measurements were obtained along the same direction using a cutoff length of 0.8 mm and a tracing length of 1.5 mm. The mean Ra value of the three measurements was subsequently calculated and used for statistical analysis. Surface morphology was examined with a JSM‐IT200 scanning electron microscope (JEOL, Germany) at magnifications of 500×, 2000×, and 5000×. Specimens were mounted on metallic stubs, sputter‐coated with gold, and imaged to assess topographical changes induced by surface treatments [25].

X‐ray diffraction (XRD) was used to evaluate the crystalline phases present on the sample surfaces. The analysis was performed using a Panalytical Empyrean X‐ray Diffractometer operating with Cu Kα radiation (*λ* = 1.54 Å). Diffraction data were collected over a 2θ range of 20°–70°, with a step size of 0.02° and a counting time of 0.6 s per step. Phase identification was conducted using search–match analysis with the International Centre for Diffraction Data (ICDD) database (Newtown Square, PA, USA). The XRD data were processed and graphically represented using OriginPro (OriginLab Corporation, Northampton, MA, USA). The software was employed for peak visualization, baseline correction, and precise diffraction pattern plotting, enabling detailed interpretation of crystallographic characteristics.

BFS was measured using a Universal Testing Machine (H50KS, Tinius Olsen Ltd., Redhill, UK) following ISO 6872:2015 [26]. Each disc was positioned on an 8 mm in diameter knife‐edge support, with the treated surface facing downward toward the support, thereby placing it on the tensile side during testing. A 3.2 mm in diameter steel ball applied a compressive load at the center at a crosshead speed of 0.5 mm/min until fracture as shown in (Figure 1). A thin rubber sheet was placed between the disc and the support to accommodate surface irregularities. The fracture load (P) in Newtons was recorded, and the BFS (σ) was calculated using the formula:

σ=−0.257⋅P⋅X−Y/d2,

where *d* is the specimen thickness, *r*_3_ is the specimen radius (5 mm), *r*_2_ is the support‐circle radius (4 mm), and *r*_1_ represents the radius of the loading configuration (1.6 mm). The geometric constants *X* and *Y* were calculated using Poisson’s ratio (*ν* = 0.25) [27, 28]. *X* and *Y* are calculated as follows:

**Figure 1:**
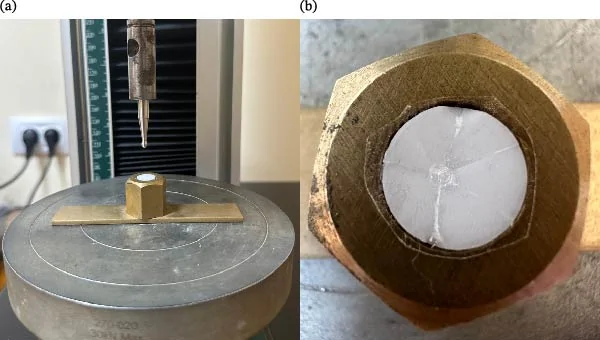
Tinius Olsen H50KS Universal Testing Machine. (a) cylindrical steel ball of 3.2 mm diameter applying load of 0.5 mm/min at center of the specimen. (b) Specimen after fracture.

X=1+νlnr2r32+1−ν2r2r32,

Y=1+ν1+lnr1r32+1−νr1r32.

Following fracture testing, the fractured fragments of each specimen were examined under a stereomicroscope (Olympus SZ61, Olympus Corporation, Tokyo, Japan) to accurately identify and count the number of fracture fragments. Fragment counting was performed by a single operator blinded to the experimental group allocation to minimize observer bias.

Data normality was confirmed using descriptive statistics, Q–Q plots, histograms, and normality tests. Parametric analyses were performed, calculating the means and standard deviations. Comparisons between the two materials were made using independent *t*‐tests with 95% confidence intervals. Comparisons among surface treatments were performed using one‐way ANOVA, followed by Bonferroni‐corrected pairwise comparisons. Two‐way ANOVA assessed the combined effects of material translucency and surface treatment on Ra, BFS, and fracture patterns, with adjusted means, standard errors, and 95% CIs reported. Statistical significance was set at *p*  < 0.05, and analyses were conducted using IBM SPSS Version 26.0. The effect of translucency and surface treatment on strength appeared to be independent (*p* = 0.42).

## 3. Results

The results revealed significant differences in surface roughness and BFS between the two zirconia types (3Y‐TZP and 5Y‐TZP) and among the various surface treatments.

For surface roughness Ra, APA with 120 µm Al_2_O_3_ produced the roughest surfaces for both zirconia types. Mean Ra rose from 0.34 ± 0.04 µm (control) to 1.09 ± 0.29 µm after APA‐120 in ZI, and from 0.40 ± 0.03 µm to 0.69 ± 0.12 µm in FX (two‐way ANOVA: treatment effect *p*‐value < 0.001; material effect *p*‐value = 0.01). Zircos E etching generated Ra values that did not differ statistically from the controls within each material (*p*‐value > 0.05). Ra of zirconia specimens showed significant variation based on translucency and surface treatment, as presented in Tables 1 and 2. Among the surface treatments, APA with 120 µm Al_2_O_3_ particles produced the roughest surfaces for both zirconia types, with 3Y‐TZP (ZI) exhibiting a higher roughness (1.09 ± 0.29 μm) than 5Y‐TZP (FX) (0.69 ± 0.12 µm; *p* < 0.001). This analysis highlighted the significant impact of both zirconia translucency and surface treatment on surface roughness, with 120 µm APA consistently yielding the roughest surfaces.

**Table 1:** Comparison of surface roughness between the two study groups across different surface treatments.

Subgroups	Translucent (ZI)	Ultratranslucent (FX)	Difference (95% CI)	*p*‐Value 1
	Mean (SD)			
Control	0.34 (0.04)^a^	0.40 (0.03)^a^	−0.06 (−0.11, −0.02)	**0.005 ^∗^**
50 µm APA	0.53 (0.08)^a^	0.55 (0.04)^b^	−0.02 (−0.09, 0.06)	0.74
120 µm APA	1.09 (0.29)^b^	0.69 (0.12)^c^	0.40 (0.13, 0.67)	**0.01 ^∗^**
Acid Etch	0.38 (0.06)^a^	0.47 (0.03)^ab^	−0.08 (−0.14, −0.03)	**0.003 ^∗^**
*p*‐Value 2	**<0.001 ^∗^**	**<0.001 ^∗^**	—	—

*Note: p*‐Value 1: Independent sample *t*‐test was used for comparison between ZI and FX groups. *p*‐Value 2: One‐way ANOVA was used for comparison between different surface treatments within each group. Different superscript letters denote statistically significant differences between subgroups using Bonferroni correction

Abbreviations: CI, confidence interval; SD, standard deviation.

^∗^Statistically significant at *p*‐value < 0.05.

**Table 2:** Two‐way ANOVA for the effect of translucency and surface treatment on surface roughness.

Variables	Translucency and subgroups	Adjusted mean (SE)	95% CI	*p*‐Value
Translucency	Translucent (ZI)	0.58 (0.02)	0.54, 0.62	**0.049 ^∗^**
	Ultratranslucent (FX)	0.52 (0.02)	0.48, 0.57	
Surface treatment	Control	0.37 (0.03)^a^	0.31, 0.43	**<0.001 ^∗^**
	50 µm APA	0.54 (0.03)^b^	0.48, 0.60	
	120 µm APA	0.89 (0.03)^c^	0.82, 0.95	
	Acid etch	0.42 (0.03)^a^	0.37, 0.48	

*Note:* Model *F* = 31.52, *p*‐value < 0.001 ^∗^, adjusted *R* ^2^: 0.78. *p*‐Value of interaction translucency × surface treatment < 0.001 ^∗^. Different superscript letters denote statistically significant differences between subgroups using Bonferroni correction.

Abbreviations: CI, confidence interval; SE, standard error.

^∗^Statistically significant at *p*‐value < 0.05.

XRD patterns of both zirconia materials showed the characteristic peaks of zirconia within the 2θ range of 20°–70°. For 5Y zirconia (FX), all surface treatment groups (control, APA‐50, APA‐120, and Zircos E) exhibited similar peak positions and comparable intensities, indicating that the applied treatments did not produce noticeable changes in the crystalline phase composition. In contrast, the 3Y zirconia (ZI) specimens demonstrated slight variations in peak intensity following APA (Figures 2 and 3). The APA‐treated groups showed the appearance of distinct monoclinic zirconia peaks (M1 and M2) at ~28° and 31.5° (2θ), indicating the presence of a tetragonal‐to‐monoclinic phase transformation, while the tetragonal peaks remained dominant. The Zircos E‐treated specimens presented diffraction patterns comparable to those of the control group, indicating that the acid‐etching treatment did not induce detectable phase transformation.

**Figure 2:**
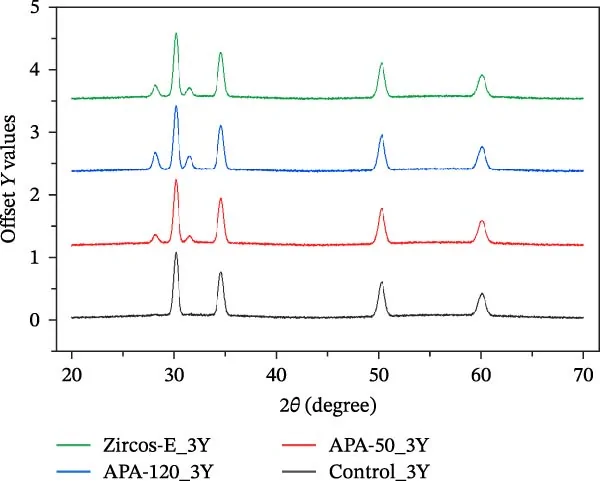
X‐ray diffraction (XRD) patterns of 3Y‐TZP after different surface treatments: untreated control, airborne‐particle abrasion with 50 µm Al_2_O_3_ (APA‐50), airborne‐particle abrasion with 120 µm Al_2_O_3_ (APA‐120), and acid etch (Zircos‐E).

**Figure 3:**
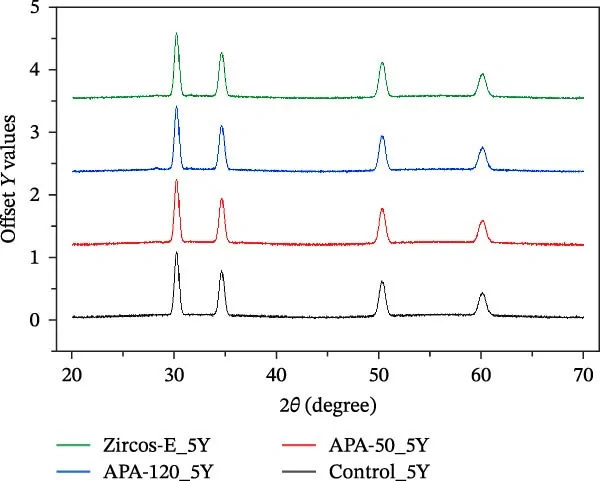
X‐ray diffraction (XRD) patterns of 5Y‐TZP after different surface treatments. All subgroups exhibited predominantly tetragonal/cubic phases, with minimal monoclinic transformation compared with 3Y‐TZP.

BFS was significantly influenced by zirconia type (*p*  < 0.001), with the translucent zirconia (ZI) exhibiting significantly higher values than the ultratranslucent zirconia (FX) across all surface treatment groups. In the control group, the mean BFS was 1059.43 ± 102.66 MPa for ZI and 500.09 ± 91.50 MPa for FX. Following surface treatment, BFS values for ZI were 931.63 ± 113.34 MPa after 50‐µm APA, 848.33 ± 231.71 MPa after 120‐µm APA, and 897.36 ± 153.17 MPa after acid etching. The corresponding values for FX were 442.71 ± 64.45 MPa, 420.29 ± 79.95 MPa, and 479.30 ± 97.09 MPa, respectively (Table 3). Within each zirconia type, differences among surface treatment groups were not statistically significant (ZI: *p* = 0.10; FX: *p* = 0.31). However, two‐way ANOVA demonstrated a significant overall effect of surface treatment (*p* = 0.03), with specimens treated using 120‐µm APA exhibiting significantly lower adjusted mean BFS than the control group, while no significant interaction was found between zirconia type and surface treatment (Table 4).

**Table 3:** Comparison of biaxial flexural strength (MPa) between the two study groups across different surface treatments.

Subgroups	Translucent (ZI)	Ultratranslucent (FX)	Difference (95% CI)	*p*‐Value 1
	Mean (SD)			
Control	1059.43 (102.66)	500.09 (91.50)	559.43 (446.18, 672.68)	**<0.001 ^∗^**
50 µm APA	931.63 (113.34)	442.71 (64.45)	488.91 (381.55, 596.28)	**<0.001 ^∗^**
120 µm APA	848.33 (231.71)	420.29 (79.95)	428.04 (226.19, 629.89)	**0.001 ^∗^**
Acid etch	897.36 (153.17)	479.30 (97.09)	418.06 (280.55, 555.58)	**<0.001 ^∗^**
*p*‐Value 2	0.10	0.31	—	—

*Note: p*‐Value 1: Independent samples *t*‐test was used for comparison between ZI and FX groups. *p*‐Value 2: One‐way ANOVA was used for comparison between different surface treatments within each group.

Abbreviations: CI, confidence interval; SD, standard deviation.

^∗^Statistically significant at *p*‐value < 0.05.

**Table 4:** Two‐way ANOVA for the effect of translucency and surface treatment on biaxial flexural strength (MPa).

Variable	Translucency and subgroups	Adjusted mean (SE)	95% CI	*p*‐Value
Translucency	Translucent (ZI)	934.19 (23.62)	886.75, 981.63	**<0.001 ^∗^**
	Ultratranslucent (FX)	460.58 (23.62)	413.14, 508.01	
Surface treatment	Control	779.71 (33.94)^a^	711.55, 847.88	**0.03 ^∗^**
	50 µm APA	687.17 (33.94)^a,b^	619.01, 755.33	
	120 µm APA	634.31 (33.94)^b^	566.15, 702.47	
	Acid etch	688.33 (31.74)^a,b^	624.57, 752.09	

*Note:* Model *F* = 30.35, *p*‐value < 0.001 ^∗^, Adjusted *R* ^2^: 0.78. *p*‐Value of interaction translucency × surface treatment = 0.42. Different superscript letters denote statistically significant differences between subgroups using Bonferroni correction.

Abbreviations: CI, confidence interval; SE, standard error.

^∗^Statistically significant at *p*‐value < 0.05.

The analysis of the number of fractured fragments showed that FX specimens exhibited greater fragmentation than that of ZI specimens across the different surface treatments. The highest number of fragments was observed in the acid‐etched FX group (6.63 ± 1.51) (Table 5 and Figure 4), indicating a more pronounced fragmentation pattern in this group. Overall, the findings demonstrated differences in fracture fragmentation according to translucency and surface treatment. The interaction between translucency and surface treatment was statistically significant for Ra but not for BFS. This suggests that while surface treatments distinctly alter the topography of ZI and FX, their impact on strength is governed more by the intrinsic properties of zirconia than by the treatment itself (Figure 5a–f).

**Figure 4:**
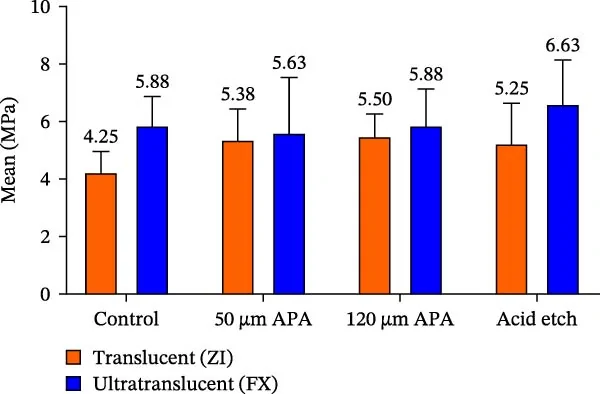
Graph showing the number of fractured fragments in the study groups.

**Figure 5:**
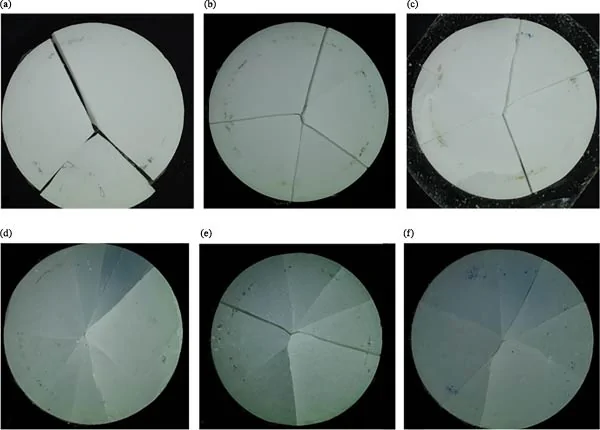
Number of fractured fragments of 3Y‐TZP (a–c) and 5Y‐TZP (d–f) zirconia discs after Zircos E etching, 50 µm APA, and 120 µm APA surface treatments respectively.

**Table 5:** Comparison of number of fractured fragments between the two study groups.

Subgroups	Translucent (ZI)	Ultratranslucent (FX)	Difference (95% CI)	*p*‐Value
	Mean (SD)			
Control	4.25 (0.71)	5.88 (0.99)	−1.63 (−2.55, −0.70)	**0.002 ^∗^**
50 µm APA	5.38 (1.06)	5.63 (1.19)	−0.25 (−1.46, 0.96)	0.66
120 µm APA	5.50 (0.76)	5.88 (1.25)	−0.38 (−1.48, 0.73)	0.48
Acid etch	5.25 (1.39)	6.63 (1.51)	−1.38 (−2.93, 0.18)	0.08

*Note:* Independent samples *t*‐test was used.

Abbreviations: CI, confidence interval; SD, standard deviation.

^∗^Statistically significant at *p*‐value < 0.05.

Scanning electron microscopy (SEM) revealed clear differences in surface morphology among the tested groups. Untreated zirconia surfaces exhibited a relatively smooth morphology with minimal irregularities (Figure 6a,e). FX specimens displayed a smoother surface with slightly smaller grains compared with ZI, which showed more densely packed grains, likely related to the higher cubic phase content of FX (5Y‐TZP). APA with 50 µm Al_2_O_3_ produced a moderately roughened surface on ZI, characterized by uniformly distributed micropits and irregular protrusions that enhanced microretentive features (Figure 6f). Increasing the particle size to 120 µm resulted in more pronounced surface damage in ZI, with deeper pits and sharp‐edged microcraters (Figure 6g). In contrast, FX subjected to the same treatment exhibited comparatively smoother surfaces with smaller and more rounded defects, suggesting a reduced abrasive impact (Figure 6b,c). These observations are consistent with the surface roughness measurements, where FX showed slightly higher roughness values (0.47 ± 0.03) than that of ZI (0.38 ± 0.06). Acid etching produced a more uniform retentive morphology with fewer sharp irregularities in both materials (Figure 6d,h). This surface pattern is consistent with the action of HF, which selectively dissolves grain boundaries and generates a homogeneous microroughened surface. Compared with APA‐treated surfaces, the smoother etched morphology may contribute to the preservation of BFS.

**Figure 6:**
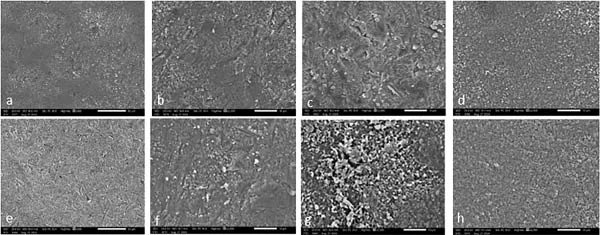
SEM images of 5Y‐TZP and 3Y‐TZP zirconia after different surface treatments: (a) 5Y‐TZP control group, (b) 5Y‐TZP treated with 50 µm APA, (c) 5Y‐TZP treated with 120 µm APA, (d) 5Y‐TZP treated with Zircos‐E etching, (e) 3Y‐TZP control group, (f) 3Y‐TZP treated with 50 µm APA, (g) 3Y‐TZP treated with 120 µm APA, and (h) 3Y‐TZP treated with Zircos‐E etching.

## 4. Discussion

The null hypothesis of this study stated that there was no significant difference in surface roughness or BFS of zirconia using the APA‐50, APA‐120, or nitric acid–HF on two types of monolithic zirconia.

The results demonstrated that the choice of surface treatment had a considerable influence on these properties, reflecting the interplay between micromechanical retention and the material’s structural integrity. Therefore, the null hypothesis was partially rejected.

APA was evaluated because it is a widely used method for enhancing zirconia surface roughness and bonding potential, despite its possible influence on phase transformation and mechanical degradation. Different APA particle sizes were included to assess their effects on the surface and structural behavior of 3Y‐TZP and 5Y‐TZP zirconia. Zircos E etching was investigated as a conservative chemical surface treatment aimed at preserving crystalline stability and flexural strength while providing surface modification [4].

Surface roughness is a critical parameter for the adhesion of restorative materials, influencing the retention and mechanical stability of restorations. In this study, APA with 120 µm Al_2_O_3_ particles resulted in the highest roughness values for both zirconia types, consistent with previous research indicating that larger abrasive particles induce more pronounced surface irregularities [29]. In this study, APA‐120 resulted in the highest surface roughness, as observed in both ZI and FX zirconia. The present findings are consistent with those of Borges et al. [30], who reported that larger APA particle sizes produce rougher zirconia surfaces, enhancing micromechanical retention while increasing the risk of reduced flexural strength due to microcrack formation. Similarly, previous studies have demonstrated that APA generates greater surface roughness than chemical etching, although the associated surface defects may compromise mechanical performance [30]. The significantly higher roughness observed in 3Y‐TZP compared with 5Y‐TZP is likely attributable to differences in their microstructure and phase composition, consistent with the findings of Falahchai et al. [ 31], who associated zirconia microstructure with its response to abrasive surface treatment.

BFS testing revealed that 3Y‐TZP consistently exhibited higher strength values across all treatments, aligning with its superior tetragonal phase content and fracture toughness [7]. Interestingly, while APA reduced flexural strength due to microcrack formation, acid etching with Zircos E appeared to strike a balance, maintaining moderate strength levels while minimizing surface damage. This reinforces the findings of Nasr et al. [23], who reported that acid etching with a nitric–HF compound effectively roughens zirconia surfaces without introducing critical defects.

SEM analysis revealed that untreated zirconia surfaces were relatively smooth, with 5Y‐TZP exhibiting a smoother surface morphology and less densely packed grains than 3Y‐TZP, reflecting differences in their microstructure and higher cubic phase content. Following surface treatment, acid‐etched specimens exhibited fewer and less pronounced surface irregularities than those of APA‐treated specimens, corroborating the observations of Zandparsa et al. [32]. In contrast, APA with 120‐µm AL_2_O_3_ particles produced the most pronounced surface alterations, consistent with the findings of Kim and Ahn [29]. The greater surface roughness observed in 3Y‐TZP compared with 5Y‐TZP is also in agreement with Jitwirachot et al. [33], who attributed these differences to variations in grain size and phase composition. However, unlike Öztürk et al. [34], who reported increased roughness associated with stress‐induced phase transformation, the present findings suggest that the applied surface treatments produced topographical modifications with limited detectable phase transformation. These commonalities and distinctions underscore the influence of material composition and treatment protocols on zirconia properties.

Pandey et al. [35] proved in his study in 2024a critical factor influencing the outcomes of flexural strength testing is specimen preparation. Variations in thickness, shape, or sintering processes can significantly impact the mechanical properties of zirconia materials [35] Inconsistent specimen preparation can lead to variability in results, potentially explaining some of the differences observed in this study between ZI and FX groups.

The XRD findings correspond well with the observed changes in the surface morphology and roughness. The limited tetragonal‐to‐monoclinic transformation detected in the 3Y‐TZP after APA is consistent with the SEM observations showing pronounced surface irregularities and microcraters produced by alumina particle impact. Mechanical surface treatments are known to induce localized stresses that promote phase transformation in tetragonal zirconia while simultaneously increasing the surface roughness. In contrast, the absence of detectable phase changes in 5Y‐TZP may be attributed to its higher cubic phase content, which provides greater structural stability and reduces susceptibility to stress‐induced transformation [36, 37]. The Zircos E‐treated specimens showed comparable XRD patterns to the control group, supporting the SEM observations of a more uniform microretentive surface with fewer sharp defects. This suggests that chemical etching may modify the surface topography without significantly affecting the crystalline phase composition of zirconia [4, 37].

The differences in mechanical performance between 3Y‐TZP and 5Y‐TZP are primarily attributable to their distinct phase compositions. The higher tetragonal phase content of 3Y‐TZP promotes stress‐induced tetragonal‐to‐monoclinic transformation, providing transformation toughening that impedes crack propagation and contributes to its superior BFS. This mechanism is supported by the XRD findings, which revealed limited tetragonal‐to‐monoclinic transformation following APA in 3Y‐TZP. In contrast, the higher cubic phase fraction of 5Y‐TZP enhances optical properties but diminishes its transformation‐toughening capability, resulting in lower BFS and the absence of detectable phase transformation after surface treatment. These findings indicate that the mechanical response of zirconia is predominantly governed by its intrinsic phase composition rather than by surface topography alone [36–38].

The findings indicate that APA with larger AL_2_O_3_ particles produces greater surface roughness but may adversely affect BFS due to the introduction of surface defects. In contrast, Zircos E acid etching provided moderate surface modification while largely preserving the mechanical properties of both zirconia types, consistent with previous studies.

Several limitations of the present study should be acknowledged. Although the effects of different surface treatments on surface roughness, BFS, and phase composition were evaluated, the adhesive performance was not assessed. Consequently, the influence of these treatments on zirconia bonding cannot be determined from the present findings. Future investigations should include bond strength testing in conjunction with surface energy, wettability, and contact angle analyses to provide a comprehensive characterization of surface treatment efficacy. Furthermore, studies involving different zirconia materials, additional surface treatment protocols, and long‐term mechanical aging are warranted. Finally, because the XRD analysis was limited to qualitative phase identification, quantitative techniques such as Rietveld refinement and the Garvie–Nicholson method were recommended to more accurately evaluate phase transformations induced by surface treatments. These findings underscore the need for material‐specific approaches to surface treatment, especially in clinical settings, where both bonding efficacy and strength are crucial for long‐term restoration success.

## 5. Conclusions

Within the limitations of this study, surface treatments significantly influenced the roughness of both zirconia materials, whereas their effect on BFS was dependent on the zirconia composition. Across all surface treatment groups, 3Y‐TZP exhibited a higher BFS than 5Y‐TZP. APA with 120 µm AL_2_O_3_ particles produced the highest surface roughness, while Zircos E etching resulted in a more conservative surface modification with limited detectable crystalline phase changes under the tested conditions. These findings emphasize the influence of zirconia composition on the response to surface treatment and highlight the importance of selecting surface treatment protocols according to material characteristics. Further studies incorporating bond strength testing, aging, fatigue loading, and quantitative phase analysis are warranted to validate the long‐term implications of these surface treatments.

**Nomenclature**
Ra: Surface roughness
BFS: Biaxial flexural strength
3Y‐TZPs: 3 mol% yttria‐stabilized tetragonal zirconia polycrystals
5Y‐TZPs: 5 mol% yttria‐stabilized tetragonal zirconia polycrystals
APA: Airborne‐particle abrasion
ZIRCOS E: Zirconia etching solution containing hydrofluoric acid (HF) and nitric acid (HNO_3_)
ZI: Ceramill Zi white zirconia
FX: Zolid FX white zirconia
CAD/CAM: Computer‐aided design/computer‐aided manufacturing
LTD: Low thermal degradation
HF: Hydrofluoric acid
MDP: Methacryloyloxydecyl dihydrogen phosphate
Al_2_O_3_: Aluminum oxide
Er: YAG: Erbium‐doped yttrium aluminum garnet.

## Author Contributions

**Yasser M. Aly**: conceptualization, methodology, investigation, writing, supervision, reviewing. **Magued H. Fahmy**: supervision, review and editing. **Ghada Ayash**: conceptualization, validation, investigation. **Ola Taha**: conceptualization, methodology, investigation, writing – original draft.

## Funding

Personal funding with no external funds.

## Ethics Statement

Ethical approval was not required for this study as it was an in vitro study and did not involve human participants, human tissues, or identifiable personal data.

## Consent

The authors have nothing to report.

## Conflicts of Interest

The authors declare no conflicts of interest.

## Data Availability

The database used and/or analyzed during this study is available from the corresponding author upon reasonable request.
